# scClustViz – Single-cell RNAseq cluster assessment and visualization

**DOI:** 10.12688/f1000research.16198.2

**Published:** 2019-03-12

**Authors:** Brendan T. Innes, Gary D. Bader

**Affiliations:** 1 Molecular Genetics, University of Toronto, Toronto, Ontario, M5S3E1, Canada; 2 The Donnelly Centre, University of Toronto, Toronto, Ontario, M5S3E1, Canada

**Keywords:** single-cell RNAseq, differential expression, functional analysis, interactive visualization, R Shiny, data sharing

## Abstract

Single-cell RNA sequencing (scRNAseq) represents a new kind of microscope that can measure the transcriptome profiles of thousands of individual cells from complex cellular mixtures, such as in a tissue, in a single experiment. This technology is particularly valuable for characterization of tissue heterogeneity because it can be used to identify and classify all cell types in a tissue. This is generally done by clustering the data, based on the assumption that cells of a particular type share similar transcriptomes, distinct from other cell types in the tissue. However, nearly all clustering algorithms have tunable parameters which affect the number of clusters they will identify in data.

The R Shiny software tool described here, scClustViz, provides a simple interactive graphical user interface for exploring scRNAseq data and assessing the biological relevance of clustering results. Given that cell types are expected to have distinct gene expression patterns, scClustViz uses differential gene expression between clusters as a metric for assessing the fit of a clustering result to the data at multiple cluster resolution levels. This helps select a clustering parameter for further analysis. scClustViz also provides interactive visualisation of: cluster-specific distributions of technical factors, such as predicted cell cycle stage and other metadata; cluster-wise gene expression statistics to simplify annotation of cell types and identification of cell type specific marker genes; and gene expression distributions over all cells and cell types.

scClustViz provides an interactive interface for visualisation, assessment, and biological interpretation of cell-type classifications in scRNAseq experiments that can be easily added to existing analysis pipelines, enabling customization by bioinformaticians while enabling biologists to explore their results without the need for computational expertise. It is available at https://baderlab.github.io/scClustViz/.

## Introduction

The development of high-throughput single-cell RNA sequencing (scRNAseq) methods, including droplet-based ( Klein *et al*., 2015; Macosko *et al*., 2015; Zheng *et al*., 2017) and multiplexed barcoding ( Rosenberg *et al*., 2018) techniques, has led to a rapid increase in experiments aiming to map cell types within tissues and whole organisms ( Ecker *et al*., 2017; Han *et al*., 2018; Regev *et al*., 2017; Saunders *et al*., 2018). The most common initial analysis of such scRNAseq data is clustering and annotation of cells into cell types based on their transcriptomes. Many workflows have been built and published around this use case ( Kiselev *et al*., 2018; Lun *et al*., 2016; Sandrine, 2016; Satija, 2018), and many clustering algorithms exist to find cell type-associated structure in scRNAseq datasets ( Li *et al*., 2017; Ntranos *et al*., 2016; Shao & Höfer, 2017; Xu & Su, 2015; Žurauskienė & Yau, 2016). This paper focuses on how to interpret the results of a scRNAseq clustering analysis performed by existing methods, specifically when it comes to selecting parameters for the clustering algorithm used and analysis of the results. This is implemented as an R Shiny software tool called scClustViz, which provides an interactive, web-based graphical user interface (GUI) for exploring scRNAseq data and assessing the biological relevance of clustering results.

Nearly all unsupervised classification (clustering) algorithms take a parameter that affects the number of classes or clusters found in the data. Selection of the appropriate resolution of the classifier heavily impacts the interpretation of scRNAseq data. An inappropriate number of clusters may result in missing rare but distinct cell types, or aberrantly identifying novel cell types that result from overfitting of the data. While there are general machine-learning-based methods for preventing overfitting, we propose a biology-based cluster assessment method; namely whether you could identify a given cluster-defined cell type *in situ* using imaging techniques based on marker genes identified, such as single molecule RNA fluorescence *in situ* hybridization (FISH). To identify marker genes and quantify the measurable transcriptomic difference between putative cell types given a clustering solution, scClustViz uses a standard differential expression test between clusters. If there are few differentially expressed genes between two clusters, then those clusters should not be distinguished from each other and over-clustering is likely. The researcher can then select a cluster solution that has sufficiently fine granularity, while still maintaining statistically separable expression of genes between putative cell types.

Once cell types are defined using the clustering method and parameters of choice, the researcher must then go through several data interpretation steps to assess and annotate these clusters and identify marker genes for follow-up experimentation. Before a final clustering result is chosen, it is important to assess the impact of technical factors on clustering. While that may have been done as part of the upstream workflow, it is helpful to see the cluster-wise distribution of technical factors such as library size, gene detection rates, and proportion of transcripts from the mitochondrial genome ( Ilicic *et al*., 2016). To annotate cell types identified by the classifier, it is helpful to see the genes uniquely upregulated per cluster, as well as assess the gene expression distribution of canonical marker genes for expected cell types in the data. Finally, novel marker genes may be identified for a cell population of interest, which requires identifying genes that are both upregulated in the cluster in question and detected sparingly or not at all in all other clusters in the experiment.

We describe scClustViz, an R package that aids this frequently encountered scRNAseq analysis workflow of identifying cell types and their marker genes from a heterogenous tissue sample. The package comprises two parts: a function to perform the differential gene expression testing between clusters for any set of clustering solutions generated by existing scRNAseq analysis workflows, and a R Shiny GUI that provides an interactive set of figures designed to help assess the clustering results, annotate cell types, and identify marker genes. The package was designed with transparency and modularity in mind to ease merging into existing workflows and sharing the results with collaborators and the public. This enables the tool to be of value to both experienced bioinformaticians developing workflows and bench scientists interpreting the results of a scRNAseq experiment.

## Methods

### Implementation

We propose a metric for assessing clustering solutions of scRNAseq data based on differential gene expression between clusters. We use the Wilcoxon rank-sum test to evaluate the statistical significance of differential gene expression between clusters. This test was selected based on the rigorous differential expression methodology review carried out by Soneson and Robinson ( Soneson & Robinson, 2018). In their testing, the Wilcoxon test had accuracy on par with that of the majority of methods tested (most methods were adequately accurate), and identified sets of differentially expressed genes similar to MAST ( Finak *et al*., 2015) and limma ( Ritchie *et al*., 2015), two popular alternatives. What little bias the Wilcoxon rank-sum test does have tends to be towards genes detected at lower rates in the data ( Soneson & Robinson, 2018), which can easily be corrected by using a detection rate filter prior to testing. In terms of power and control of type I error rate, the Wilcoxon test was less powerful than more advanced methods, with a false discovery rate (FDR) more conservative than expected. However, unlike some more complicated tests, the Wilcoxon test is compatible with parallel processing of testing calculations to increase computation speed. Ultimately, the simplicity of the Wilcoxon test made it appealing for default use in this tool, as it is understood by most users, is fast to compute and is available in base R. Alternatively, given the wide variety and constant growth of scRNAseq-specific differential gene expression tests, scClustViz can use the results of any test method that returns measures of effect size and statistical significance.

Two measures of effect size of differential gene abundance are reported by scClustViz: difference in detection rate (dDR) and gene expression ratio (logGER, log2 gene expression ratio). Detection rate refers to the proportion of cells from each cluster in which the gene in question was detected (per cluster gene detection rate). The concept of detection rate in scRNAseq data stems from the low per-cell sensitivity and minimal amplification noise of droplet-based assays. Since there is a correlation between gene expression magnitude and per cluster gene detection rate, the detection rate is a meaningful quantification of gene expression. Furthermore, it is suitable for identifying genes that uniquely “mark” certain cell populations, as such marker genes should be undetected outside of the cells they mark.

**Figure 1.**
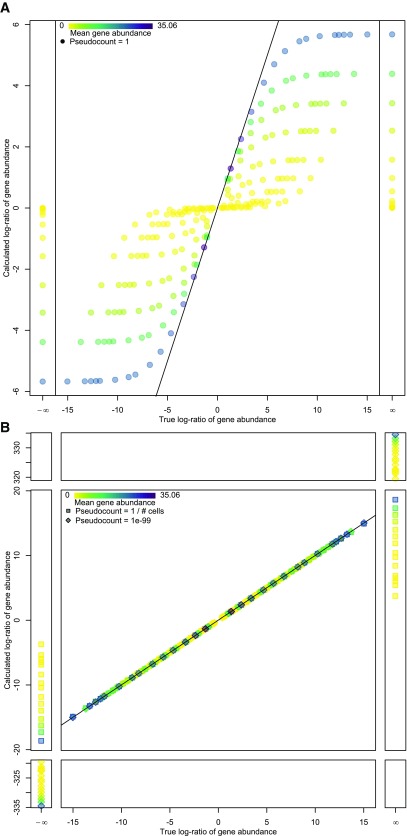
Mean and log gene expression ratio (logGER) calculations are affected by selection of the pseudocount used to prevent divide-by-zero errors. **A**. A scatter plot comparing true logGER (x-axis) with logGER calculated with a pseudocount of 1 (y-axis) for pairwise comparisons from a simulated scRNAseq data set where the mean abundance of a single gene varied from zero to 50 across 15 clusters. Points are coloured by the mean gene abundance of the comparison, with darker being larger. The black line denotes equality between x- and y- axes. With a pseudocount of 1, the magnitude of logGER is compressed at both ends relative to true logGER, and the magnitude of this compression is inversely correlated with gene abundance in the clusters being compared. **B**. Same plot comparing true logGER with logGER calculated with pseudocounts of 1e-99 (diamonds) and 1 / # of cells (squares). Calculated logGER are very close to true logGER when using smaller pseudocounts (as denoted by the black line). When using a very small pseudocount of 1e-99, the magnitude of logGER values are over 300 when comparing a cluster with zero gene abundance (division-by-zero resulting in a true logGER magnitude of infinity). This is far from the range of other logGER values. An alternative is to set the pseudocount to the smallest possible “step” in count-based data (1 / # of cells) to prevent magnitude compression of logGER calculations caused by using a pseudocount of 1, while keeping division-by-zero values within the range of the data. Code to generate this figure is available in the scClustViz folder of the R library under paperFigs/Fig1.R

Log gene expression ratio (also known as log fold change) is a measure of effect size that considers both magnitude of gene expression as well as detection rate, as it is the ratio of mean gene abundance between two cell clusters. However, due to the sparsity of scRNAseq data, some clusters may not contain any cells in which a certain gene was detected. It is thus necessary to add a pseudocount to the logGER calculations to prevent divide-by-zero errors and the resulting logGER magnitudes of infinity. As exemplified in Figure 1, the choice of pseudocount impacts logGER results. A pseudocount of 1 is commonly used in the field of transcriptomics but creates two problems when used on the low abundance values common to droplet-based scRNAseq data. Since a value of 1 is a considerable fraction of small count data, adding 1 to all counts tends to compress the magnitude of the gene expression ratio in a manner that inversely correlates with the magnitude of abundances being compared ( Figure 1a). As a result, not only is the calculated logGER less than true logGER, but this compression of true logGER is more pronounced when at least one side of the comparison has values near zero. Using a small pseudocount such as 10 ^-99^, on the other hand, results in logGER values being very close to their true value, rather than suffering from the compression caused by the pseudocount of 1 ( Figure 1b). The problem with this is that comparisons with zero result in very high magnitude logGER values, well outside the range of the rest of the results. If zero counts of a transcript in a cell library truly represented that gene not being expressed at all in that cell (i.e. if high-throughput single-cell RNAseq experiments were exquisitely sensitive), then this wouldn’t be a problem, since the true expression ratio would be infinitely large. However, zero counts are better interpreted to mean that transcripts for the gene in question were not detected in that cell. Given the relatively poor sensitivity of current high-throughput scRNAseq technology on a per cell basis, this does not necessarily mean that the gene was not expressed. Thus, it would be better if logGER values for comparisons with zero were reasonably close in magnitude to the rest of the results. To accomplish this, we use a pseudocount representing the smallest possible “step” in the count-based data, set to the reciprocal of the number of cells in the data. This is sufficiently small as to not compress logGER magnitudes, while keeping comparisons with zero reasonably close to the range of potential logGER values. In scClustViz, the reported logGER values are ratios of log-mean gene abundance calculated using the reciprocal of the number of cells in the data (the smallest possible “step” in the cDNA count) as the pseudocount.

Three different sets of differential gene expression results are reported by scClustViz. These are the results of two sets of hypothesis tests: each cluster versus the rest of the data combined (calculated by the function CalcDEvsRest), and all pairwise comparisons between clusters (calculated by the function CalcDEcombn). These comparisons are made using the Wilcoxon rank-sum test, with false discovery rate controlled using the method of Benjamini and Hochberg ( Benjamini & Hochberg, 1995). Genes are included in the test if they pass a detection rate threshold (default is 10%) in at least one of the pair of clusters tested. In the case of both sets of tests, the results can be substituted with those of another statistical method by adding its results to the sCVdata object outlined below.

The first set of genes reported by scClustViz are those that are differentially expressed between each cluster and the rest of the data combined (referred to as DE vs Rest in the Shiny interface). This is not used to assess clustering results but may be visualized by the user to identify distinguishing genes for that cluster, although this will only be valuable if there is enough heterogeneity in the data to identify differential genes. Though this represents an unbalanced comparison, the non-parametric nature of the Wilcoxon rank-sum test makes it robust to such imbalances.

The second is referred to as marker genes. These are genes that are significantly positively differentially expressed in a cluster in pairwise comparisons with every other cluster (at a default FDR of 5%). This is taken from the results of the pairwise comparisons outlined above and returned by the function DEmarker. This method is one of the two sets of differential gene expression results used in scClustViz to quantify cluster granularity. It ensures that there are marker genes for all clusters that are unique to each cluster, given all other clusters in the data.

The third set of genes reported is calculated by comparing each cluster to its nearest neighbouring cluster, and represents the other way cluster granularity is quantified by scClustViz. By ensuring there is at least one positively differentially expressed gene (default FDR of 5%) between each set of neighbouring clusters, this metric enforces the requirement for having statistically separable clusters, which is less restrictive than requiring unique marker genes per cluster. Nearest neighbours are clusters with the fewest differentially expressed genes between them, as calculated above. These are also taken from the results of the pairwise comparisons outlined above and returned by the function DEneighb.

To quickly compare multiple clustering solutions in the user interface, the above differential gene expression tests and other cluster-wise gene expression statistics are precomputed for each cluster solution. The results are stored as a named list containing entries for each cluster solution. The precomputed results for each cluster solution are stored as a novel S4 object class, sCVdata.

To support quick display of the various figures in the user interface, other cluster-wise gene statistics are calculated. Detection rate (DR) is the proportion of cells in a cluster in which a given gene has a non-zero expression value. Mean detected gene expression (MDGE) is the mean of the normalized transcript counts for a gene in the cells of the cluster in which that gene was detected. And mean gene expression (MGE) is the mean normalized transcript count for a gene for all cells in the cluster. These are stored as a named list of dataframes in a slot in sCVdata.

Both pairwise and one versus all differential expression test results are similarly stored in slots of sCVdata (DEvsRest and DEcombn). For the results of comparisons between a cluster and the rest of the data, each named list element contains a data frame with logGER for all genes, and p-value and FDR results for all tested genes. For pairwise comparisons between clusters, each named list element contains a data frame with logGER and dDR for all genes, and p-value and FDR results for all tested genes. List elements are named with cluster names, separated by a dash for pairwise comparisons.

The sCVdata object also stores the results of silhouette analysis, a metric for assessing the contribution of each cell to cluster cohesion and separation ( Rousseeuw, 1987). This is included in the visualization as a complementary metric for cluster solution assessment. Finally, user-defined parameters pertaining to calculations on the input data are also stored as a slot in sCVdata, supporting replicability.

The package was built in R v3.5.0 ( R Core Team, 2018). The R Shiny interactive web page generating tool (shiny v1.1.0) was used to generate the scClustViz user interface ( Chang *et al*., 2018). Silhouette plots are generated using the R package cluster v2.0.7-1 ( Maechler *et al*., 2018). Colour-split dots for plotting use code from the R package TeachingDemos v2.10 ( Snow, 2016). Colour scales with transparency use the R packages scales v1.0.0, viridis v0.5.1, and RColorBrewer v1.1-2 ( Garnier, 2018; Neuwirth, 2014; Wickham, 2017).

### Operation

The scClustViz tool is available as an R package from GitHub, with usage details and example code available on the website. The typical usage requires one setup step prior to running the visualization to precompute and save the differential gene expression testing results. Once setup is complete, the user can quickly view and easily share the results of their analysis.

Setup is done using the function *CalcAllSCV*, which takes as input the user’s scRNAseq data object and a data frame of cluster assignments where each variable refers to a different cluster solution. Currently scClustViz supports both the Bioconductor SingleCellExperiment class ( Lun & Risso, 2017) and Seurat class ( Butler *et al.*, 2018; Satija *et al.*, 2015). This function also takes optional arguments describing the state of the data and customizing testing thresholds. To calculate means of log-normalized data accurately, the function needs to know the log base and pseudocount used in the normalization. In most cases, gene expression data is transformed in log base 2, though Seurat uses the natural log. Most log-normalization methods add a pseudocount of 1 to avoid log-zero errors. As such, the function defaults to expecting log2-normalized data with a pseudocount of 1. The function also allows the user to set the gene detection rate threshold for inclusion in differential gene expression testing, defaulting to 10%.

Since this step may be time-consuming with many cluster solutions to test, the function includes an option to stop testing cluster solutions once differential gene expression between nearest neighbouring clusters has been lost. In order to do this, the function tests cluster solutions in order of increasing numbers of clusters and ensures that all nearest neighbouring cluster pairs (as determined by number of differentially expressed genes in pairwise tests) have at least one significant comparison. As such, the user may indicate the false discovery rate threshold for determining significance, which defaults to 5%.

Alternatively, the differential gene abundance testing and cluster overfitting determination can be incorporated into an existing analysis pipeline. This can be done by iteratively clustering with increasing resolution and calling CalcSCV after each clustering step. CalcSCV generates an sCVdata object for a single cluster resolution, and is called by CalcAllSCV to generate the list of sCVdata objects needed to run the Shiny interface. By checking for differential expression between nearest neighbouring clusters, this can be used to automatically stop generating cluster solutions once differential expression between clusters is lost.

The resulting list of sCVdata objects and input scRNAseq data object should be saved to disk as a single compressed RData file prior to viewing them in the GUI. This is done to ensure that setup is a one-time process, and to simplify sharing and reproducibility of analyses. The function *runShiny* launches the R Shiny instance with the interactive data figures in the R integrated development environment (IDE) or a web browser. It loads the data from a file and has optional arguments to specify the annotation database and marker genes for expected cell types. The annotation database is used to find gene names to improve clarity of some figures and expects a Bioconductor AnnotationDbi object such as org.Mm.eg.db for mouse or org.Hs.eg.db for human. Finally, if passed a named list of canonical marker genes for expected cell types in the data, scClustViz will automatically generate cluster annotations (labels). This is done by assigning each cluster to the cell type with the top aggregate rank of gene expression for its marker genes. More in-depth and unbiased methods for assigning cell type identities to clustering results exist ( Crow *et al*., 2018; Kiselev *et al.*, 2017), so this is meant more as a convenience option for labelling purposes than a definitive automatic cluster annotation method.

System requirements for this tool will depend heavily on the data set in question, since the data will have to be loaded into memory, and the memory footprint of scRNAseq data depends on the number of cells being analysed. However, in all tests loading objects from Seurat into scClustViz, the saved objects after the setup and differential expression testing steps were smaller than the original Seurat object. It is thus safe to assume that scClustViz will run on the computer on which the data set in question was analysed. For the data from the MouseCortex package, the largest data set (E15, containing nearly 3000 cells) uses less than 1.2GB of memory. Opening Shiny apps can be difficult in some computing environments, especially remote R sessions to servers without browsers or rendering capabilities. There are options in the Shiny runApp function to help troubleshoot these situations, and these are accessible from the runShiny function in scClustViz.

## Use cases

To demonstrate the convenience of sharing analysed data with scClustViz, the MouseCortex package was built with data from a recent publication exploring the development of the mouse cerebral cortex using scRNAseq ( Yuzwa *et al*., 2017). A tutorial for building similar R data packages calling scClustViz as the visualization tool can be found on the scClustViz website.

The MouseCortex package contains the four data sets published in the paper, and a wrapper function for *runShiny* that loads each data set with the appropriate arguments. The embryonic day 17.5 data set (opened by the command *viewMouseCortex(“e17”)*) will be used to demonstrate the purpose of the various figures in scClustViz and highlight its role in identifying a core gene set expressed in the neurogenic stem cell population of the cerebral cortex in the next sections. All figures from this point on were generated in the scClustViz Shiny app and saved using the “Save as PDF” buttons.

### Clustering solution selection

**Figure 2.**
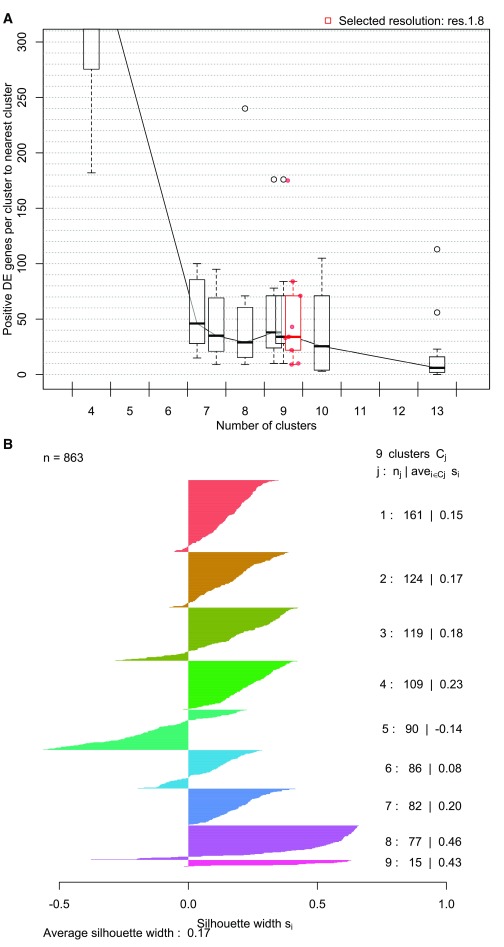
Interactive figures to assess clustering solutions. **A**. Boxplots representing number of differentially expressed genes between neighbouring clusters for each cluster resolution. For each cluster at a specific resolution, the number of positively differentially expressed genes to its nearest neighbouring cluster is counted, and those counts are represented as a boxplot. The boxplots are arranged along the x-axis to reflect the number of clusters found at that resolution. Highlighted in red is the cluster resolution currently selected in the interface. This figure has been zoomed using the interactive interface to make it clear that at the selected resolution there is more than one differentially expressed gene between neighbouring clusters. The number of marker genes per cluster and average silhouette widths can be similarly viewed with the scClustViz interface. **B**. Silhouette plot for the selected cluster resolution. A horizontal bar plot where each bar is a cell, grouped by cluster. Silhouette width represents the difference between mean distance to other cells within the cluster and mean distance to cells in the neighbouring cluster. Distance is Euclidean in reduced dimensional (generally PCA) space. Positive values indicate that the cell is closer to cells within its cluster.

The first step in the post-clustering workflow is to assess the results of the various clustering parameterizations used. scClustViz uses a combination of differential gene expression between clusters and silhouette analysis for this. Differential gene expression is used as a metric in two ways: the number of positively differentially expressed genes between a cluster and its nearest neighbour, and the number of marker genes (positively differentially expressed vs. all other clusters in pairwise tests) per cluster. In Figure 2a, differential expression to the nearest neighbouring cluster is represented as a series of boxplots per cluster resolution, arranged on the x-axis to indicate the number of clusters in each boxplot. The highlighted boxplot indicates the currently selected cluster from the pulldown menu in the user interface. Both differential expression-based metrics can be visualized this way by switching the metric used, via the interface.

When a cluster resolution is selected, its silhouette plot is rendered to add another method of cluster assessment ( Figure 2b). A silhouette plot is a horizontal bar plot where each bar is a cell, grouped by cluster. The width of each bar, referred to as silhouette width, represents the difference between mean distance to other cells within the cluster and mean distance to cells in the neighbouring cluster. Distance is Euclidean in the reduced dimensional space used in clustering (this is generally PCA space, and is pulled from the input data object based on a user-defined parameter). Positive values indicate that the cell is closer to cells within its cluster. It is worth noting that the dimensions returned by methods such as PCA are not equally meaningful, since each explains a different proportion of the variance in the data, while Euclidean distance treats them all equally. This can be addressed by weighting the PCs by variance explained, a method implemented in newer versions of Seurat ( Butler *et al*., 2018). To prevent unexpected results caused by assuming a PC weighting option in upstream analysis, the silhouette plot in scClustViz does not reweight PCs, so users are encouraged to consider this when interpreting this plot.

Once the user has chosen the appropriate cluster solution, they can click the “View clusters at this resolution” button to proceed to in-depth exploration and visualization of the results. They can also save this as the default resolution for future sessions. If a cluster resolution is saved as default, a file specifying the saved resolution will be generated in the same directory as the input data (or an optional output directory). Specifying a separate output directory is useful when the input data is part of a package, as in MouseCortex. If the same output directory is specified the next time the command is run, all saved data in that directory will be reloaded in the app.

### Data set and cluster metadata inspection

**Figure 3.**
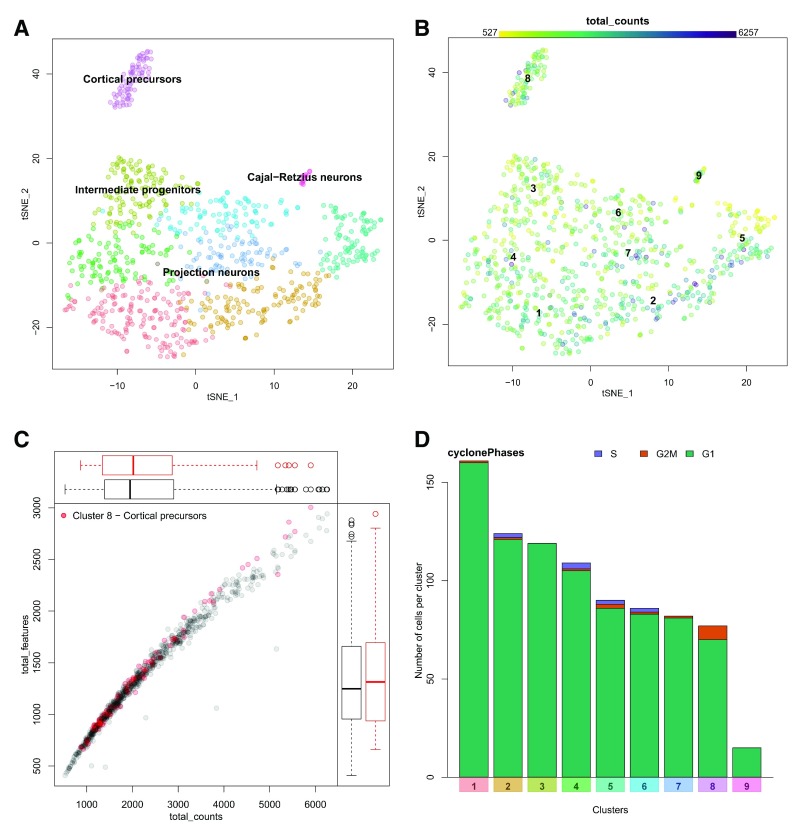
Visualizations of the data and its metadata. **A**. A 2D projection of cells in gene expression space (frequently a tSNE plot) is coloured by cluster. Clusters can be labelled by number, or automatically annotated as seen here. **B**. An example of a metadata overlay on the tSNE plot. The library size (number of transcripts detected) per cell is represented by colour scale, where darker cells have larger library sizes. **C**. Metadata can be represented as a scatter plot. The relationship between number of unique genes detected (total features – y-axis) and library size (total counts – x-axis) is shown here. The cells from the selected cluster (cluster 8, cortical precursors) are highlighted in red. **D**. Categorical metadata is represented as a stacked bar plot showing the number of cells contributing to each category per cluster. This plot shows predicted cell cycle state, with G1 phase in green, G2/M in orange, and S phase in purple.

In this section, the user can explore the data set as a whole. The first panel, Figure 3a shows a two-dimensional representation of cells in gene expression space. This is generally a tSNE or UMAP plot, and is pulled from the input data object based on a user-defined parameter ( McInnes & Healy, 2018; van der Maaten & Hinton, 2008). The cells are coloured by cluster and can be labelled by cluster number or automatically annotated with a predicted cell type based on known marker genes for expected cell types passed to *runShiny*. The user can select any cluster for downstream exploration by clicking on a cell from that cluster in this plot. This will highlight the cluster in other plots in the interface. Since we are interested in identifying marker genes for the precursor cell population, we may click on cluster 8 (purple) to select it for downstream analysis.

The distribution of various cellular metadata can be visualized in Figure 3b. Metadata is selected from a pulldown menu and is represented as colours on the cells in the 2D projection. In this manner the user can inspect the impact of technical artefacts such as gene detection rate, library size, or cell cycle stage on the clustering results. Numeric metadata can also be assessed as a scatter plot, where the axes can be defined by selecting from pulldown menus. Figure 3c shows the relationship between number of genes detected and library size per cell for both the data set as a whole and the selected cluster. The cells from cluster 8, a cortical precursor cluster, were selected in the previous plot and are thus highlighted in red here. The cluster 8 cells are similar to other cells in the data, thus do not seem to be biased by the measures visualized in this plot. If this was not the case, we may want to consider investigating confounding variables in the normalization process. For example, many authors have noted that gene detection rate is often strongly correlated with the first few principal components, and can unduly influence clustering results ( Finak *et al*., 2015; Risso *et al*., 2018). There are a few ways to handle this, from simply excluding those principal components, or explicitly normalizing for those factors when scaling the data (as implemented in Seurat), to including the offending technical variables as covariates in more complex dimensionality reduction (i.e. ZINB-WaVE) or differential expression testing (i.e. MAST) models. While those specialized analyses are outside the scope of this tool, it is important to be able to visualize these technical factors in the analysed data to assess the efficacy of the chosen correction method.

Categorical metadata is represented as a stacked bar plot in Figure 3d, as either absolute counts or relative proportions. Here we see that by E17.5 the cortical precursors of cluster 8 are not predicted to be actively in the cell cycle using the *cyclone* method ( Scialdone *et al*., 2015). This fits expectations from known developmental biology, since neurogenesis is nearly complete by this stage, and the stem cell population that persists into adulthood is thought to enter quiescence around E15.5 ( Fuentealba *et al*., 2015). For demonstration purposes, we will continue to focus on cluster 8, which is predicted to form the adult neurogenic stem cell population in the cerebral cortex. We will aim to identify marker genes for these cells.

### Differentially expressed genes per cluster

**Figure 4.**
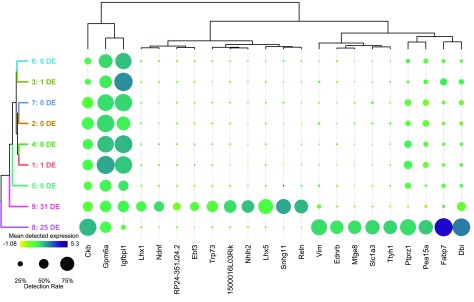
Visualizing differential gene expression. A dot plot showing the relative expression of a subset of marker genes (x-axis) across all clusters (y-axis). A dot plot is a modified heatmap where each dot encodes both detection rate and average gene expression in detected cells for a gene in a cluster. Darker colour indicates higher average gene expression from the cells in which the gene was detected, and larger dot diameter indicates that the gene was detected in greater proportion of cells from the cluster. Cluster colours are indicated for reference on the left side of the plot. Cluster numbers are also indicated on the left side, along with the number of differentially expressed genes in each cluster. The genes included can be changed to reflect those differentially expressed per cluster when compared to the rest of the data set as a whole (i.e. the tissue), the nearest neighbouring cluster, or marker genes unique to that cluster. This figure shows marker genes per cluster. The number of differentially expressed genes contributed per cluster can also be adjusted, here set to 10.

Once the user is satisfied that their cluster solution is appropriate and unaffected by technical factors, the next step in data interpretation is to determine the cellular identity of each cluster by its gene expression profile. The differential expression tests done prior to running the visualization assist with this by highlighting the most informative genes in the data set. In a sufficiently heterogeneous data set, differential expression between a cluster and the rest of the data can be useful for identifying genes that uniquely define a cluster’s cellular identity. A more conservative form of this is the identification of marker genes – those genes that are significantly positively differentially expressed in all pairwise tests between a cluster and all other clusters. This highlights genes expected to be found at a significantly higher expression in this cluster than anywhere else in the data. Finally, there is the testing between each cluster and its nearest neighbour to highlight local differences in expression. Each of these sets of differentially expressed genes can be presented as a dot plot comparing clusters, as seen in Figure 4. A dot plot is a modified heatmap where each dot encodes both detection rate (by dot diameter) and average gene expression in detected cells (by dot colour) for a gene in a cluster. Here up to the top ten marker genes per cluster are shown, but both the type of differential expression test used to generate the gene set and the number of differentially expressed genes contributed per cluster can be adjusted using the interactive interface. At this point in the analysis, it is also possible to download any of these differential gene expression results as tab-separated value files for further analysis, by selecting the cluster of interest and differential expression type and clicking “Download gene list”. This may be of value if the user is using this platform to share the data online, or with those who would prefer not to use R for further analysis. In this dot plot, we can see the top 10 marker genes for our putatively quiescent cortical precursor cell population (cluster 8) include known marker genes for cortical radial precursors ( *Fabp7*, *Slc1a3*, *Ptprz1*, and *Vim*), a known marker for adult neural stem cells ( *Dbi*), as well as novel marker genes for this population ( *Mfge8*, *Ttyh1*, *Pea15a*, and *Ednrb*) ( Yuzwa *et al*., 2017). The dot plot format also shows us that while *Ckb* and *Gpmgb* are significantly positively differentially expressed in cluster 8 relative to all other clusters, they are still detected in high proportions in all clusters, and thus would not be optimal marker genes.

### Gene expression distributions per cluster

**Figure 5.**
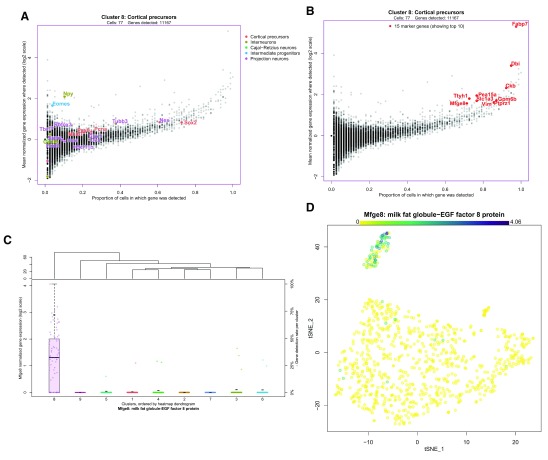
Exploring cluster-wise gene expression. **A**. A scatter plot representing gene expression in the highlighted cluster, the cortical precursor cluster 8. The x-axis represents the proportion of cells from that cluster in which a gene is detected (more than zero transcript counts), and the y-axis is the mean normalized abundance from cells in which the gene was detected. The cell type marker genes are highlighted, indicating that this cluster was classified as cortical precursors based on the high relative expression of both *Sox2* and *Pax6*, as well as *Nes* and *Cux1* (markers for both cortical precursors and projection neurons). **B**. The same scatter plot is shown with the top 10 marker genes for cluster 8 highlighted, though the user can choose other differentially expressed gene sets from the heatmap, or search for genes of interest using the interface. The identity of any point can be determined by clicking on it in the interface. **C**. Boxplots comparing the expression of a gene of interest across all clusters. Clusters are arranged on the x-axis based on the cluster dendrogram generated for the dot plot above ( Figure 4), and normalized transcript count for the gene of interest ( *Mfge8*, in this case) is represented on the y-axis. The dots on each boxplot represent the individual data points, gene expression per cell. The black dash is an optional indication of the gene detection rate per cluster, as indicated on the y-axis on the right side. This figure shows that *Mfge8* may be a marker of cortical precursors. **D**. Gene expression overlaid on the cell projection. Gene expression is represented by a colour scale on the cells of the two-dimensional projection, where darker indicates higher expression. Clusters can be optionally labelled by number or annotation. This figure shows the distribution of *Mfge8* expression in the dataset.

To more closely inspect the gene expression of an individual cluster, scClustViz presents gene expression data per cluster as a scatter plot with the proportion of cells from that cluster in which a gene is detected (more than zero transcript counts) on the x-axis, and mean normalized transcript count from cells in which the gene was detected on the y-axis, as seen in Figure 5a. This visualization method helps separate the contribution of zeros from the mean gene expression value, since like the dot plot it separates magnitude of gene expression from gene detection rate. It also highlights the strong relationship between magnitude of gene expression and likelihood of detection in droplet-based single-cell RNAseq data, since the trend goes from the plot’s bottom left (genes have low expression and are rarely detected) to top right (genes have high expression and are detected often). In this figure, the cortical precursor cluster 8 is shown, but the user can select the cluster shown from a pulldown menu in this panel as well. There are three ways to highlight various genes in this plot. First, the genes passed as known marker genes for expected cell types can be highlighted in colours corresponding to their cell type, if a marker gene list is defined by the user ( Figure 5a). This figure indicates that this cluster was classified as cortical precursors based on the high relative expression of both *Sox2* and *Pax6*, as well as *Nes* and *Cux1* (markers for both cortical precursors and projection neurons). In Figure 5b, the plot shows differentially expressed genes, specifically the genes contributed by this cluster to the dot plot shown immediately above in the app ( Figure 4). Thus, by changing the differential gene set or number of genes in the heatmap, the user can also adjust the genes highlighted in this scatter plot. Finally, the user can search for genes manually by entering a list of gene symbols or using a regular expression in the search box below the figure. To identify and compare gene expression for any point in this figure, the user can click on the corresponding data point.

Clicking on a data point in the figure above will generate a series of boxplots comparing gene expression for the selected gene across all clusters ( Figure 5c). Since the above scatter plot can be crowded, all genes near the clicked point are shown in a pulldown menu, so that the user can select their gene of interest. Alternatively, the gene(s) entered in the search box in the previous panel can be used to populate the pulldown list for selecting the gene of interest for this figure. By comparing gene expression across clusters, it is easier to assess the utility of putative marker genes. Here we see that *Mfge8* is expressed nearly exclusively in cluster 8, with rare detection in any other clusters. This suggests that *Mfge8* may be effective for identifying the cells of this cluster *in situ*. In fact, both fluorescence *in situ* hybridization for *Mfge8* and immunohistochemistry for its protein lactadherin showed specificity for the cortical precursor cells in the embryonic mouse brain, as well as the B1 neural stem cells of the adult ventricular/subventricular zone ( Yuzwa *et al*., 2017).

Finally, the user can directly plot the expression of a gene or genes of interest on the tSNE plot to better visualize the distribution of gene expression in the data set, as shown in Figure 5d. Genes are selected by entering gene symbols or a using a regular expression and selecting the matching gene symbols from a dropdown list. Gene expression is represented by a colour scale on the cells of the two-dimensional projection. If multiple genes are selected, the maximum gene expression value per cell is shown. This serves as another way of highlighting the specificity of *Mfge8* for the cortical precursor cells in this data set.

### Cell set comparisons

**Figure 6.**
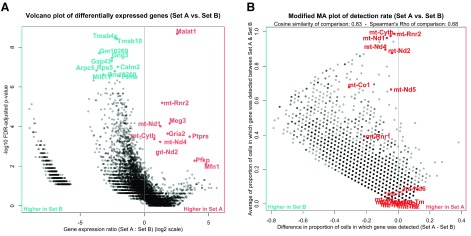
Comparing manually defined sets of cells. **A**. A volcano plot showing log-ratio of gene expression between cell sets on the x-axis, and differential gene expression significance score (-log10 FDR) on the x-axis. Set A here is a subset of cluster 5 with low library sizes (< 1500 counts per cell), while set B is the subset of cluster 5 with high library sizes (> 1500 counts per cell). Highlighted are the top differentially expressed genes upregulated in set A (red) and set B (blue). **B**. An MA-style plot showing difference in gene detection rate between set A and set B on the y-axis, and average gene detection rate across sets on the x-axis. The vertical line is at zero difference in detection rate. Highlighted in red are genes from the mitochondrial genome, which are generally used as markers of damaged cells in single-cell RNAseq analyses.

The final feature of scClustViz is the ability to generate volcano and MA plots comparing gene statistics for any two clusters, or any two sets of cells specified by the user ( Figure 6a). This is useful for two reasons. First, such detailed investigations of differences between clusters may help identify cell types or classify their relationships. It may also reveal systematic differences in gene expression data between two sets of cells that could indicate a technical or biological confounding factor. Volcano plots show relationships between effect size and statistical significance for sets of differential gene expression comparisons between clusters. MA plots (also known as Tukey’s mean-difference plot or Bland-Altman plot) show differences between samples comparing the log-ratio of gene expression between samples to the mean gene expression across those samples. We modify the traditional MA plot by showing the mean on the y-axis and difference on the x-axis to maintain visual consistency with volcano plots. We further expand this plot’s utility by giving the user the option of viewing the difference and average of all three gene statistics used in scClustViz: mean gene expression, mean detected gene expression, and detection rate. Furthermore, the user can manually select sets of cells to compare, and scClustViz will calculate differential gene expression statistics between the selected cells and the remaining cells in the data, and between sets of selected cells. Once the calculations are complete, the resulting comparison is represented as a separate “cluster solution” and can be explored in all the figures of scClustViz. These results can be saved to disk by clicking “Save this comparison to disk” when selecting it in the pulldown menu for cluster solution selection. Any saved comparisons will be loaded along with the data any time *runShiny* is run.

In Figure 6 we’re investigating a potential technical artefact in the data, specifically the poor cohesion of cluster 5 as seen in the silhouette plot in Figure 2b. This poor cohesion could be due to the differences in library size within the cells of the cluster, as seen in Figure 3b. To investigate this, the cell selection tool in scClustViz was used to select the cells of cluster 5 with low library sizes (Set A, < 1500 UMIs per cell) and those with high library sizes (Set B, > 1500 UMIs per cell). After running the differential gene expression calculations, we can view the differentially expressed genes between the sets in the dot plot or volcano plot ( Figure 6a). Set B seems to have more positively differentially expressed genes, which may be due to improved gene detection rate from higher library sizes. This can be seen in Figure 6b, where an MA-style plot showing difference in detection rate vs average detection rate across sets is shown. Most genes are more detected in the set with larger library sizes (set B), which might be expected, since more transcripts detected correlates with higher average transcript counts per gene. Clicking on a gene in this figure has the same functionality as the scatter plot in Figure 5; it will be selected for viewing in the boxplot above ( Figure 5c). Using this, we noticed that genes from the mitochondrial genome were seemingly unaffected by the difference in library sizes, as they tended to fall near zero difference in detection rate. To highlight this, we searched for all genes from the mitochondrial genome using the search tool, which allowed us to highlight them here. If cells are damaged and leaking cytoplasm, they are likely to have smaller library sizes as they lose mRNA. However, since RNA from the mitochondrial genome is sequestered in a separate organelle, they are less likely to lose those transcripts ( Ilicic *et al*., 2016). We can see evidence for this in the cells of cluster 5 with small library sizes, since the detection rate of their mitochondrial genes is unchanged. While this data set was filtered to remove cells with higher than average mitochondrial gene transcript proportions, including that metric in the metadata would allow for tuning of the threshold used. Since these cells have both low library sizes and higher relative detection rate of mitochondrial transcripts, it is safe to assume they are damaged cells and remove them from the analysis.

## Conclusion

We developed scClustViz to aid in the annotation of cell types and identification of marker genes from scRNAseq data. It provides both a metric for cluster assessment based on inter-cluster differential gene expression, as well as a convenient user interface for accomplishing this analysis and interpretation task. Using differential gene expression to assess clustering solutions ensures that the results are suited to addressing the relevant biological task of identifying cell types and their marker genes. The user interface is also focused specifically on this task by generating publication quality figures and providing analyses that help the user determine the appropriate number of clusters, identify cell types, and highlight genes unique to those cell types. There are other user interfaces available for the analysis of scRNAseq data ( Rue-Albrecht *et al*., 2018; Zhu *et al*., 2017). However, scClustViz fills a niche between existing GUIs, which are either very user-friendly for non-technical users, at the cost of the ability to customize analysis, or very powerful and customizable, at the cost of providing a simple framework for accomplishing a common analysis task. The one-time setup step for scClustViz also simplifies data sharing, as it generates a file that can be shared for viewing by anyone using R. Data sharing can be made more user-friendly by building an R data package with a wrapper function calling scClustViz, as seen in the use case outlined in this paper. Building such a package is a quick process, and a tutorial is available on the scClustViz website. scClustViz is available at https://baderlab.github.io/scClustViz/ as free, open source software under the permissive MIT open source license.

## Data availability

The example dataset used is available as an R package: https://github.com/BaderLab/MouseCortex

Archived code at time of publication: https://doi.org/10.5281/zenodo.2582093 ( Innes, 2018a)

Licence: MIT

## Software availability

scClustViz is available from: https://baderlab.github.io/scClustViz/

Source code is available from GitHub: https://github.com/BaderLab/scClustViz

Archived source code at time of publication: https://doi.org/10.5281/zenodo.2582090 ( Innes, 2018b)

Licence: MIT
