# Supplementary material for: scClustViz – Single-cell RNAseq cluster assessment and visualization
Source: F1000Res. 2019 Mar 12;7:ISCB Comm J-1522. Originally published 2018 Sep 21. [Version 2] doi: 10.12688/f1000research.16198.2 (PMC6456841; doi:10.12688/f1000research.16198.2)
Supplement: Peer review history

# Peer review: Reviewer response for version 2

**DOI:** 10.5256/f1000research.20249.r45602

**Published:** 2019-04-09

**Authors:** Michael Steinbaugh

**Affiliations:** 1 Constellation Pharmaceuticals, Cambridge, MA, USA

I read through the revisions and approve of the changes.

I have read this submission. I believe that I have an appropriate level of expertise to confirm that it is of an acceptable scientific standard.

# Peer review: Reviewer response for version 2

**DOI:** 10.5256/f1000research.20249.r45603

**Published:** 2019-03-20

**Authors:** Martin Hemberg, Tallulah Andrews

**Affiliations:** 1 Wellcome Genome Campus, Wellcome Sanger Institute, Hinxton, UK; 2 Wellcome Sanger Institute, Hinxton, UK

The authors have addressed all of our concerns. In particular, the improvements in importing objects worked seamlessly so we are happy to recommend for indexing.

We have read this submission. We believe that we have an appropriate level of expertise to confirm that it is of an acceptable scientific standard.

# Peer review: Reviewer response for version 1

**DOI:** 10.5256/f1000research.17688.r38632

**Published:** 2018-10-10

**Authors:** Michael Steinbaugh

**Affiliations:** 1 Constellation Pharmaceuticals, Cambridge, MA, USA

**Strengths**

**Interactive Shiny interface. **scClustViz enables researchers to work interactively with their single-cell RNA-seq data. The Shiny interface available in this package is excellent. I found it to be very intuitive and easy to use. Cluster identification and marker analysis is time consuming, and scClustViz helps accelerate these steps by enabling a user to quickly generate and save informative plots.

**Cluster and marker visualizations.** The suite of plots offered in this package are robust and informative. They all appear to render quickly, even for relatively large data sets. In particular, the silhouette plot approach (Figure 3B) is novel, and I like the ability to view the cluster and maker dimensional reduction plots side-by-side (Figure 4). Dot plots are a great way to identify cell-type specific markers, and the interactive tool available in the package works well (Figure 5).  

**Weakness**

**logGER pseudocount calculations. **The explanation of differential expression implementation needs improvement (see Methods section), and the functions are not documented in the text. The authors mention that they employ a small pseudocount calculation approach (e.g. using 10 ^-99^), which results in "logGER values being very close to their true value". More evidence is needed to support this claim, and I would like to see additional rationale as to why this is approach is preferable to other published methods that address dropout/zero count inflation (e.g. ZINB-WaVE, MAGIC) ^1^- ^2^.

**Wilcoxon rank-sum test.** Additionally, as mentioned in the text, the Wilcoxon rank-sum test isn't as powerful and doesn't control the false discovery rate as well as some other published methods ^3^. In particular, edgeR, DESeq2, limma, scde, and MAST are validated differential expression callers in use in other single-cell analysis packages that are viable alternatives to Wilcoxon ^4^- ^5^- ^6^- ^7^- ^8^- ^9^. An option to use pre-calculated values inside the package would be a nice addition.

**Reproducible code.** While the interactivity provided by the Shiny interface is excellent and user-friendly for visualization, GUI-based data analysis is often difficult to reproduce. I would like to see scriptable, exported versions for all of the tools available in the package, so that a single-cell marker analysis can be run start-to-finish using scClustViz in a reproducible manner.

**Recommendations**

**Take advantage of object-oriented programming. **The authors mention in the text that scClustViz relies upon a "small custom list" of data generated using either the `readFromSeurat()` or `readFromManual()` functions, and that this approach "reduces the risk of unexpected inputs". I disagree with this statement, and recommend that the authors switch from an unstructured list to an S4 class object. Additionally, the paper doesn't describe what is stored in this list in detail. The S4 class system is documented in detail on the Bioconductor website, and is used by most popular single-cell RNA-seq analysis tools. S4 classes enable validity checks (see `validObject()`) and backwards compatibility support for legacy objects created with older versions of the package (see `updateObject()`). 

**Add native SingleCellExperiment support.** The authors provide a function for importing data from Seurat (`readFromSeurat()`), but currently don't provide a simple coercion method for the popular `SingleCellExperiment` class container.

**Add unit testing. **I noticed that the package doesn't currently have any code coverage with unit tests. I strongly recommend adding these checks against a minimal dataset. In particular, the testthat package (http://testthat.r-lib.org) is recommended. 

**Improve text labels. **The gene marker labels on some plots are illegible because they are superimposed. The ggrepel package (https://cran.r-project.org/package=ggrepel) may help improve the legibility of plots with gene labels.

**Technical issues**

**Unable to launch Shiny browser in a remote R session.** The example MouseCortex Shiny data package runs correctly on machines where a browser instance can be launched. I tested this on multiple local environments (Linux, macOS, Windows) and on a remote RStudio server. However, it fails to launch on some remote high-performance computing (HPC) environments from the R command line. In some cases this can potentially be fixed with `runApp(launch.browser = FALSE)`, but it's unclear to me whether a user can easily run the `viewMouseCortex("e11")` example in a remote R session without RStudio. This may be an edge case, but many R users work primarily on remote environments, so it's worth mentioning this potential limitation in the text. Here is the error message that can occur:

 ```

 Listening on http://127.0.0.1:3899

 xdg-open: no method available for opening 'http://127.0.0.1:3899'

 ```

**Shiny console warnings.** A number of warnings appear in my R console when running the example Shiny datasets. For reference, here are a few I can see in my log when viewing the MouseCortex example dataset:

 ```

 Warning: Error in if: argument is of length zero

   [No stack trace available]

 Warning: Error in tapply: arguments must have same length

   [No stack trace available]

 Warning: Error in switch: EXPR must be a length 1 vector

   [No stack trace available]

 ```

I have read this submission. I believe that I have an appropriate level of expertise to confirm that it is of an acceptable scientific standard, however I have significant reservations, as outlined above.

## References

[1] RissoDPerraudeauFGribkovaSDudoitSVertJ: A general and flexible method for signal extraction from single-cell RNA-seq data. *Nature Communications*.2018;9(1) : 10.1038/s41467-017-02554-5 10.1038/s41467-017-02554-5 PMC577359329348443

[2] van DijkDSharmaRNainysJYimKKathailPCarrAJBurdziakCMoonKRChafferCLPattabiramanDBierieBMazutisLWolfGKrishnaswamySPe'erD: Recovering Gene Interactions from Single-Cell Data Using Data Diffusion. *Cell*.2018;174(3) : 10.1016/j.cell.2018.05.061 716-729.e27 10.1016/j.cell.2018.05.061 29961576PMC6771278

[3] SonesonCRobinsonMD: Bias, robustness and scalability in single-cell differential expression analysis. *Nat Methods*.2018;15(4) : 10.1038/nmeth.4612 255-261 10.1038/nmeth.4612 29481549

[4] RobinsonMDMcCarthyDJSmythGK: edgeR: a Bioconductor package for differential expression analysis of digital gene expression data. *Bioinformatics*.2010;26(1) : 10.1093/bioinformatics/btp616 139-40 10.1093/bioinformatics/btp616 19910308PMC2796818

[5] LoveMIHuberWAndersS: Moderated estimation of fold change and dispersion for RNA-seq data with DESeq2. *Genome Biol*.2014;15(12) : 10.1186/s13059-014-0550-8 550 10.1186/s13059-014-0550-8 25516281PMC4302049

[6] FinakGMcDavidAYajimaMDengJGersukVShalekAKSlichterCKMillerHWMcElrathMJPrlicMLinsleyPSGottardoR: MAST: a flexible statistical framework for assessing transcriptional changes and characterizing heterogeneity in single-cell RNA sequencing data. *Genome Biol*.2015;16: 10.1186/s13059-015-0844-5 278 10.1186/s13059-015-0844-5 26653891PMC4676162

[7] RitchieMEPhipsonBWuDHuYLawCWShiWSmythGK: limma powers differential expression analyses for RNA-sequencing and microarray studies. *Nucleic Acids Res*.2015;43(7) : 10.1093/nar/gkv007 e47 10.1093/nar/gkv007 25605792PMC4402510

[8] KharchenkoPVSilbersteinLScaddenDT: Bayesian approach to single-cell differential expression analysis. *Nat Methods*.2014;11(7) : 10.1038/nmeth.2967 740-2 10.1038/nmeth.2967 24836921PMC4112276

[9] ButlerAHoffmanPSmibertPPapalexiESatijaR: Integrating single-cell transcriptomic data across different conditions, technologies, and species. *Nat Biotechnol*.2018;36(5) : 10.1038/nbt.4096 411-420 10.1038/nbt.4096 29608179PMC6700744

# Response

**Published:** 2019-03-04

**Authors:** Brendan Innes

**Affiliations:** University of Toronto

Dr. Steinbaugh,

 Thank you for your detailed review of our manuscript, and your helpful suggestions for improving the software. We address your comments below.

***logGER pseudocount calculations.*** *The explanation of differential expression implementation needs improvement (see Methods section), and the functions are not documented in the text.*

 We’ve clarified our differential expression testing methods.

*The authors mention that they employ a small pseudocount calculation approach (e.g. using 10-99), which results in "logGER values being very close to their true value". More evidence is needed to support this claim, and I would like to see additional rationale as to why this is approach is preferable to other published methods that address dropout/zero count inflation (e.g. ZINB-WaVE, MAGIC).*

 Log gene ratio (also referred to as log fold change) is a common way of reporting effect size for differential gene abundance tests. These calculations are independent of the statistical test, but play a role in the interpretation of results, and we expressed a concern that the traditional method of calculating these ratios (using a pseudocount equal to 1) was underrepresenting the true magnitude of effect size due to the small abundances common to droplet-based scRNAseq data. Using a very small pseudocount (e.g. 10-99) is not appropriate either. Instead, we recommend using a pseudocount representing the smallest possible “step” in the count-based data, set to the reciprocal of the number of cells in the data. We generated a simulated dataset that more clearly represents the problem, comparing the three pseudocount options we discuss, with results plotted in Figure 1. The analysis used to generate the data is available as an R script installed with scClustViz and found in the R library path under scClustViz/paperFigs/Fig1.R.

***Wilcoxon rank-sum test.*** *Additionally, as mentioned in the text, the Wilcoxon rank-sum test isn't as powerful and doesn't control the false discovery rate as well as some other published methods. In particular, edgeR, DESeq2, limma, scde, and MAST are validated differential expression callers in use in other single-cell analysis packages that are viable alternatives to Wilcoxon. An option to use pre-calculated values inside the package would be a nice addition.*

 Great point. We’ve now included a simple way of passing results from other differential expression callers into the workflow (replacing default values in the scClustViz data object).

***Reproducible code.*** *While the interactivity provided by the Shiny interface is excellent and user-friendly for visualization, GUI-based data analysis is often difficult to reproduce. I would like to see scriptable, exported versions for all of the tools available in the package, so that a single-cell marker analysis can be run start-to-finish using scClustViz in a reproducible manner.*

 This is an excellent idea. scClustViz now exports all functions used for both calculation of the data presented in the Shiny interface, and those used to generate the figures available in the interface.

***Take advantage of object-oriented programming.*** *The authors mention in the text that scClustViz relies upon a "small custom list" of data generated using either the `readFromSeurat()` or `readFromManual()` functions, and that this approach "reduces the risk of unexpected inputs". I disagree with this statement, and recommend that the authors switch from an unstructured list to an S4 class object. Additionally, the paper doesn't describe what is stored in this list in detail. The S4 class system is documented in detail on the Bioconductor website, and is used by most popular single-cell RNA-seq analysis tools. S4 classes enable validity checks (see `validObject()`) and backwards compatibility support for legacy objects created with older versions of the package (see `updateObject()`).*

 This was a welcome suggestion and forms the basis for our major update to scClustViz. As outlined in the updated manuscript, the setup step prior to running the Shiny interface now interfaces with existing S4 objects of the SingleCellExperiment and Seurat classes, and stores results of its calculations in a custom S4 class “sCVdata”. This should make loading analyses into scClustViz simpler for the user, there is less opportunity for unexpected data formats, and error messages are now clearer. The one aspect of this suggestion we did not implement was the backwards compatibility support. If we change the class structure in the future, we will do so, but since Drs. Andrews and Hemberg identified a statistical error in our previous version, we opted to not support backwards compatibility to prevent the propagation of that error into users results going forward.

***Add native SingleCellExperiment support.*** *The authors provide a function for importing data from Seurat (`readFromSeurat()`), but currently don't provide a simple coercion method for the popular `SingleCellExperiment` class container.*

 scClustViz now interfaces with the SingleCellExperiment class.

***Add unit testing.*** *I noticed that the package doesn't currently have any code coverage with unit tests. I strongly recommend adding these checks against a minimal dataset. In particular, the testthat package (http://testthat.r-lib.org) is recommended.*

 Testing has been added for all functions performing calculations. Inspired by a recent blog post, we have also added integration of Travis CI and Codecov as in issue in our github tracker to incorporate in the near future.

***Improve text labels.*** *The gene marker labels on some plots are illegible because they are superimposed. The ggrepel package (https://cran.r-project.org/package=ggrepel) may help improve the legibility of plots with gene labels.*

 This was not implemented in the first version of scClustViz because all plots where gene labels are present are clickable, allowing the user to disambiguate overlapping labels. However, this doesn’t help when users export their figures for the purpose of static presentations, so this was a valuable suggestion. We’ve now developed a function (spreadLabels2) for base R graphics inspired by ggrepel and spreadLabels that attempts to eliminate label overlap while keeping labels as close to their data points as possible.

***Unable to launch Shiny browser in a remote R session.*** *The example MouseCortex Shiny data package runs correctly on machines where a browser instance can be launched. I tested this on multiple local environments (Linux, macOS, Windows) and on a remote RStudio server. However, it fails to launch on some remote high-performance computing (HPC) environments from the R command line. In some cases this can potentially be fixed with `runApp(launch.browser = FALSE)`, but it's unclear to me whether a user can easily run the `viewMouseCortex("e11")` example in a remote R session without RStudio. This may be an edge case, but many R users work primarily on remote environments, so it's worth mentioning this potential limitation in the text.*

 An ellipsis argument to pass options to runApp is now included in the runShiny function (and wrapper functions calling it for published datasets), which may help the user troubleshoot in computing environments that don’t easily run Shiny apps. This is now mentioned in the system requirements section in the manuscript.

***Shiny console warnings.*** *A number of warnings appear in my R console when running the example Shiny datasets.*

 This seems to be a side-effect of Shiny’s real-time evaluation of functions. There are times when a function returns an error because an input it depends on is being calculated by another function. Shiny returns this as a warning, but once the calculation is complete, the dependent function can run error-free, so these warnings aren’t pertinent. This may be addressed by adding some internal checks to ensure dependent functions run only when their dependencies have been satisfied. We have added this to our github issue tracker to address in the future.

 Thank you again for all these valuable suggestions, which have improved the robustness and user-friendliness of scClustViz.

# Peer review: Reviewer response for version 1

**DOI:** 10.5256/f1000research.17688.r38629

**Published:** 2018-10-10

**Authors:** Martin Hemberg, Tallulah Andrews

**Affiliations:** 1 Wellcome Genome Campus, Wellcome Sanger Institute, Hinxton, UK; 2 Wellcome Sanger Institute, Hinxton, UK

In this manuscript Innes and Bader present scClustViz, an R package for interactive assessment and visualization of unsupervised clustering methods for scRNA-se data. The topic is very timely as unsupervised clustering is one of the most important applications of scRNA-seq. Nevertheless, it is a challenging problem and despite several different software tools being available, it is still not possible to fully automate the process. Thus, having a method to facilitate this analysis that can be run on the output of other clustering methods is potentially very useful.

 Major Comments:

 1. Installing scClustViz was straightforward and easy. However, I then had some issues running it. Using a Seurat object from another project where we are analyzing the data, I got the following error:

 DE_for_scClustViz <- clusterWiseDEtest(data_for_scClustViz,exponent=exp(1))

 [1] “”

 [1] “”

 [1] “Calculating all DE stats for  res.0.8”

 [1] “”

 [1] “Calculating cluster gene summary statistics”

 [1] “-- Gene detection rate per cluster --”

   |++++++++++++++++++++++++++++++++++++++++++++++++++| 100% elapsed = 01m 39s

 [1] “-- Mean detected gene expression per cluster --”

   |++++++++++++++++++++++++++++++++++++++++++++++++++| 100% elapsed = 01m 36s

 [1] “-- Mean gene expression per cluster --”

   |++++++++++++++++++++++++++++++++++++++++++++++++++| 100% elapsed = 01m 24s

 [1] “”

 [1] “Calculating DE vs tissue with 16 clusters”

 [1] “-- logGER calculations --”

   |++++++++++++++++++++++++++++++++++++++++++++++++++| 100% elapsed = 25m 39s

 [1] “-- Wilcoxon rank sum calculations --”

   |++++++++++++++++++++++++++++++++++++++++++++++++++| 100% elapsed = 11m 20s

 [1] “”

 [1] “Calculating marker DE with 120 combinations of clusters”

   |                                                  | 0 % ~calculating  Error in intI(i, n = x@Dim[1], dn[[1]], give.dn = FALSE) :

  invalid character indexing

 It may be that there are some issues with the Seurat object that I used as input, but the unspecific nature of the error message makes it very hard to troubleshoot.

 I then tried to run it using an SingleCellExperiment object. Here, the instructions were less clear and it required some fiddling around before I came up with the following lines of code for preparing the dataset, the FACS sorted lung data from the Tabula Muris:

 logcounts(mySCE) <- log2(counts(mySCE) + 1)

 clusterAssignments <- grepl("^cell_type1",colnames(colData(mySCE)))

 mySCE <- plotPCA(mySCE, return_SCE=T, draw_plot=F)

 mySCE <- plotTSNE(mySCE, return_SCE=T, draw_plot=F)

 data_for_scClustViz <- readFromManual(nge=logcounts(mySCE),

                                       md=colData(mySCE)[,!clusterAssignments],

                                       cl=as.character(colData(mySCE)[,clusterAssignments]),

                                       dr_clust=reducedDim(mySCE,"PCA"),

                                       dr_viz=reducedDim(mySCE,"tSNE"))

 DE_for_scClustViz <- clusterWiseDEtest(data_for_scClustViz,

                                        # Stop once DE is lost between nearest neighbouring clusters

                                        testAll=FALSE,

                                        # Normalized data is in log2 space

                                        exponent=2,

                                        # Pseudocount of 1 was added to log-normalized data

                                        pseudocount=1,

                                        # False discovery rate threshold of 1%

                                        FDRthresh=0.01,

                                        # Use difference in detection rate to filter genes for testing

                                        threshType="dDR",

                                        # Genes with at least 15% detection rate difference will be tested

                                        dDRthresh=0.15

                                        )

 save(data_for_scClustViz,DE_for_scClustViz,file="for_scClustViz.RData")

 This allowed me to get the shiny interface started, but there were several error messages appearing (most frequently "object 'dr_viz' not found") and I was not able to explore the different functionalities that had been highlighted in the manuscript. This may have been due to the fact that there was only one clustering present in the SingleCellExperiment object. However, if this is the case, then I think that the error messages should be more informative and scClustViz ought to do a better job of handling this special case. Thus, I was unable to explore the different functionalities that were highlighted in the manuscript.

 2. The multiple filters used by scClustViz is in general a good idea since it is not clear what is the best way of defining DE genes. However, filtering by changes in detection rate invalidates the multiple testing correction, genes could be filtered by expression level or detection rate but not difference in detection rate. The authors should comment on this complication

 Minor comments:

 1. Figure 3A plot would be better presented as a grouped scatterplot since the number of values per box is small.

 2. Figure 4 D colours in legend do not match colours in figure.

We have read this submission. We believe that we have an appropriate level of expertise to confirm that it is of an acceptable scientific standard, however we have significant reservations, as outlined above.

# Response

**Published:** 2019-03-04

**Authors:** Brendan Innes

**Affiliations:** University of Toronto

Drs. Andrews and Hemberg,

 Thank you very much for your careful and helpful comments on this manuscript and software.

 Your first major concern related to errors when trying to run the setup step. In the new version of scClustViz this has been addressed by using a formal S4 class object to store the results of the analysis. This object class includes built-in validity checking, so unexpected inputs are caught early and reported with meaningful error messages. In keeping with this, scClustViz now accesses data directly from SingleCellExperiment and Seurat S4 objects, which should reduce the number of unexpected inputs, as they have their own validity checking and return consistent data structures.

 Your second major concern was that filtering for difference in detection rate prior to differential expression testing invalidates the multiple testing correction, and you suggest filtering for detection rate instead. This is a very good point, since any filtering of hypotheses using a feature that correlates with the hypothesis being tested invalidates the assumption of uniform p-value distributions in FDR correction. We have addressed this by adopting the detection rate filter commonly used in the field, where genes must be detected above a certain rate (10% is our default) in at least one of the clusters being compared. This filtering method continues to protect against making comparisons with low-abundance genes that the Wilcoxon rank-sum tests may be biased towards. As a result, we have removed the section of the manuscript comparing the previous proposed filtering methods, and the previous figure 2.

 You also suggest using a grouped scatterplot instead of boxplot for Fig3a (now Fig2a). The reason we avoided a grouped scatterplot in this case was because some cluster solutions may result in the same number of clusters and thus overlap on the x-axis. This would make it challenging to display a grouped scatterplot without causing confusion. We’ve opted to compromise by showing the data points for the selected cluster solution only.

 Finally, you noted that the legend in Fig4d (now Fig3d) has an incorrect legend. We couldn’t identify the error, so if it persists we’d be happy to correct it.

 Thank you again for identifying important points to address. We hope these corrections will allow you to use the software without further difficulty.
